# The rate of smoking in patients who have suffered from ACLR graft rupture

**DOI:** 10.1002/jeo2.70237

**Published:** 2025-04-24

**Authors:** W. P. Yau

**Affiliations:** ^1^ Department of Orthopaedics and Traumatology, School of Clinical Medicine, Li Ka Shing Faculty of Medicine The University of Hong Kong Hong Kong SAR China

**Keywords:** ACLR, graft rupture, smoking

## Abstract

**Purpose:**

The purpose of this study was to investigate the rate of smoking in patients who suffered from anterior cruciate ligament reconstruction (ACLR) graft rupture. It was hypothesised that there would be no difference in the ACLR graft rupture rates between smokers and non‐smokers.

**Methods:**

A retrospective study was conducted on patients who underwent primary ACLR using hamstring autograft at the author's institute between 2007 and 2021. Patients with unknown smoking status were excluded. All included patients received post‐operative magnetic resonance imaging for assessment of graft integrity. The rate of graft rupture was reported. A case–control study was performed to determine the relationship between smoking and ACLR graft rupture by matching age, sex, chronicity of tear, diameter and type of ACLR graft, Grade 3 pivot‐shift, type of ACLR and concomitant anterolateral ligament reconstruction between smokers and non‐smokers.

**Results:**

A total of 495 primary ACLRs were included. The patients were aged 27.2 ± 8.2 years. There were 397 men and 98 women, with a smoking rate of 20%. The average follow‐up was 78 ± 48 months. 16.2% of smokers, compared to 10.1% of non‐smokers, suffered from graft rupture. The median yearly rate of graft rupture was 2 per 100 ACLRs (interquartile range [IQR] = 2.4) for smokers, compared to 1 per 100 ACLRs (IQR = 0.6) for non‐smokers. One hundred seventy‐two patients were matched. Smokers were found to have a higher risk of suffering from graft rupture compared to non‐smokers in the case–control matching analysis (odds ratio = 2.6, 95% confidence interval = 1.01–6.63).

**Conclusion:**

In a cohort of 495 primary ACLRs operated using hamstring autograft with a 20% smoking rate, the rates of ACLR graft rupture and revision ACLR at a mean follow‐up of 6.5 ± 4 years were 11.3% and 8.3%, respectively. Smoking was associated with an increased risk of graft rupture in a case–control matching subgroup analysis.

**Level of Evidence:**

Level IV.

**Abbreviations**
ACL: anterior cruciate ligament
ACLR: anterior cruciate ligament reconstruction
CI: confidence interval
EUA: examination under anaesthesia
HS: hamstring
IKDC: International Knee Documentation Committee
IQR: interquartile range
MRI: magnetic resonance imaging
RCT: randomised controlled trial
TAS: Tegner activity scale

## INTRODUCTION

Smoking is a significant risk factor that affects health problems globally [15]. Smokers are more susceptible to inferior outcomes after anterior cruciate ligament reconstruction (ACLR) than non‐smokers [23]. Smoking is associated with a higher risk of post‐operative infection [4, 24] and thromboembolic disease [4]. Additionally, smokers are more likely to experience persistent pain [17], lower function scores [17, 18], greater anterior knee translation [17, 18] and a lower chance of returning to pre‐injury sport [17].

Smoking is known to delay the ligamentization of the ACLR graft, as shown in post‐operative magnetic resonance imaging (MRI) [8]. However, there is controversy surrounding the association of smoking with ACLR graft rupture [2, 4, 11, 16, 19, 20, 23]. Cancienne et al. reported in a matching study that included 13,358 ACL reconstructions, that the rate of subsequent ipsilateral or contralateral ACLR was higher in smokers compared to non‐smokers, with an odds ratio (OR) of 1.7 [4]. At a median follow‐up of 140 months, Lu et al. reported that a history of smoking was a predictor for an increased risk of graft rupture [20]. On the other hand, two recent systematic reviews suggested that smoking is neither associated with ACLR graft rupture [11] nor contralateral knee ACL injury [10].

Graft rupture after ACLR is influenced by a large number of covariates [8, 12, 13, 14, 21, 25, 26, 27, 28, 30, 31, 33], including patient factors [4, 8, 14, 16, 26, 31], surgeon factors [14, 25, 26, 30] and disease factors [13, 14]. The most suitable methodology to study the association between smoking and ACLR graft rupture is a prospective cohort study or a retrospective case–control study. However, there is a need to control the large number of covariates known to affect ACLR graft rupture, which many studies reporting the relationship between smoking and ACLR graft rupture fail to do [1, 11, 16, 18, 19, 22].

The purpose of this study was to investigate the rate of smoking in patients who suffered from ACLR graft rupture. The rate of graft rupture in smokers and non‐smokers was reported. The association of smoking with graft rupture was assessed in a case–control matching study, taking into consideration most of the known pre‐operative and intra‐operative covariates that affect ACLR graft rupture. It was hypothesised that there would be no difference in the ACLR graft rupture rates between smokers and non‐smokers.

## METHODS

The current study was approved by the local ethics committee at the author's institute (Document number: UW 24‐412), with a waiver of the need to obtain informed consent from the patients.

A retrospective study using prospectively collected data was conducted. Patients were included if they (i) underwent primary ACLR using hamstring (HS) autograft at the author's institute between July 2007 and December 2021, and (ii) were skeletally mature, as confirmed by the pre‐operative X‐ray of the involved knee. Patients were excluded if (i) their smoking status was not known, (ii) they did not receive a post‐operative reassessment MRI, or (iii) they had a follow‐up of less than 2 years.

### Collection of demographic data

Patients were assessed in a pre‐operative assessment clinic 1 week before the scheduled surgery. Demographic data, including smoking status and medical information such as the Tegner activity scale (TAS), International Knee Documentation Committee (IKDC) subjective score and physical examination findings of both knees, were obtained. Regarding the subjective IKDC score and TAS, patients were asked to complete a self‐administered questionnaire, which included the subjective IKDC form, pre‐injury and pre‐operative TAS, laterality of the involved knee and time of injury. Information regarding age and sex was obtained through the citizens' identification system of the author's city. Medical information, such as smoking status and physical examination findings, were obtained in a pre‐operative assessment consultation by a team of orthopaedic surgeons, including the author of this manuscript. Information regarding body height and body weight was gathered by a team of physiotherapists. The information was prospectively documented using a standard research documentation form designed for other studies conducted at the author's institute.

### Definition of smoking

Smoking was defined as the use of conventional cigarettes and did not include the consumption of e‐cigarettes or heated tobacco products [34]. The definition of smoking was adopted from the regular citywide census conducted by the local government once every 5 years [5, 6]. At the time when the prospective data collection form was designed in 2007, the definition of smoking provided by the local government's census report did not include e‐smoking [5]. In the current study, the smoking status was recorded at the time of ACLR. Smokers were defined as patients who had smoked before the time of ACLR. Smokers included current daily smokers, current non‐daily smokers and ex‐smokers. Ex‐smokers were defined as those who had stopped smoking before ACLR, regardless of the time of quitting. Non‐smokers were defined as those who had never smoked at the time of ACLR.

### Surgical procedure

ACLRs were performed by two surgeons, including WPY. Patients were placed under general anaesthesia. An examination under anaesthesia (EUA) was performed. A Grade 3 pivot shift phenomenon was defined as a locked subluxation [32]. Medial HSs were harvested from the ipsilateral knee. Diagnostic arthroscopy was carried out, and concomitant intra‐articular pathology, including meniscus lesions, was treated accordingly. Both single‐bundle and double‐bundle reconstructions were options for ACLR that were performed during the period of inclusion. The type of ACLR performed depended on the research projects conducted at the time of operation and the choice of the patient. Concomitant anterolateral ligament reconstruction was performed in selected cases from 2013 onwards.

### Rehabilitation and follow‐up

The rehabilitation protocol was standardised for patients who received primary ACLRs and included a 9‐month rehabilitation programme at the same rehabilitation centre. Bracing was not applied. Patients were advised to practice partial weight‐bearing walking in the first 3 weeks after surgery. The programme included an initial 3‐month closed‐chain training, followed by open‐chain training in the subsequent 3 months. Agility training was initiated 7 months post‐operation. Patients were advised not to return to pivoting sports within the first 9 months after ACLR.

The patient was followed up in a designated ACLR clinic once every 3 months in the first year after surgery, and then annually. IKDC subjective scores were applied, and physical examination findings were recorded at each follow‐up.

### Reassessment MRI

Reassessment MRIs were scheduled for all patients as part of the follow‐up protocol and were performed in the second year after the index surgery unless patients refused. An additional MRI would be arranged if the patient experienced repeated injury, raising suspicion of ACLR graft rupture or other intra‐articular injury. Graft rupture was diagnosed through MRI or diagnostic arthroscopy.

### Statistics

Descriptive statistics were reported. Statistical analysis was performed using SPSS version 29. Independent *t* tests were used to compare continuous data, while chi‐square tests were used to compare categorical data. Statistical significance was assumed if *p* < 0.05. If a statistically significant association was found by the chi‐square test, the OR and the corresponding 95% confidence interval (CI) were reported.

### Subgroup analysis—Case–control matching

A subgroup analysis was conducted using a one‐to‐one case–control matching methodology to investigate the association between smoking and ACLR graft rupture. Factors considered during the case–control matching were (i) age, (ii) sex, (iii) time between injury and surgery, (iv) presence of a Grade 3 pivot shift phenomenon during EUA, (v) type of ACLR, including single‐bundle and double‐bundle ACLR, (vi) diameter of ACLR graft and (vii) concomitant anterolateral ligament reconstruction. Propensity scores were generated with respect to smoking status for continuous data, such as age, time between injury and surgery, and the diameter of the ACLR graft. Matching of the propensity score was performed with a Mahalanobis distance of 0.01, while matching of the categorical covariates was performed by exact matching of the items. The number of successfully matched pairs was reported. The association between smoking and graft rupture was studied using a chi‐square test. The potential difference in survivorship between smokers and non‐smokers was assessed using Kaplan–Meier survivorship analysis.

## RESULTS

**Figure 1:**
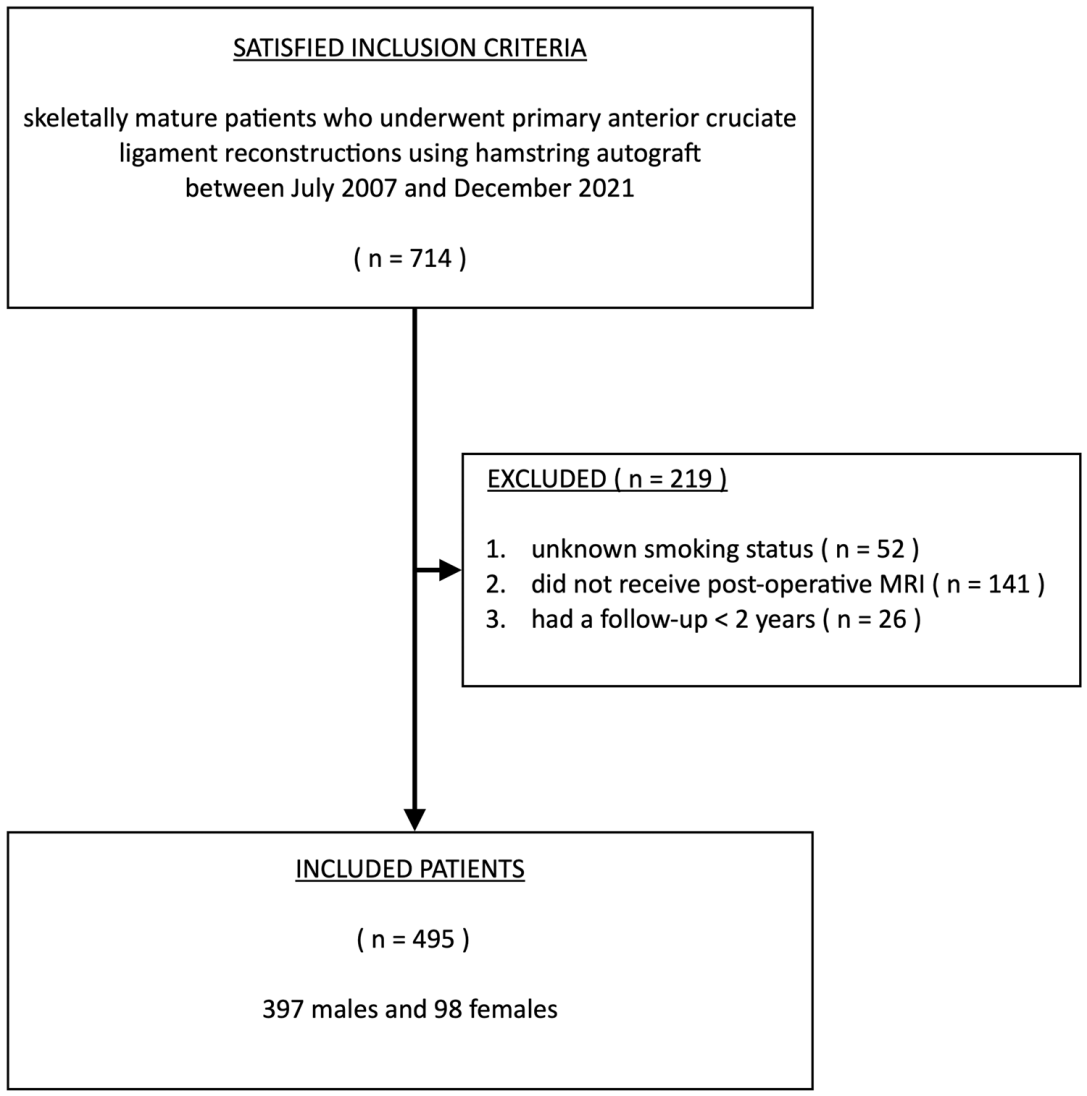
Patient selection. MRI, magnetic resonance imaging.

A total of 714 primary ACLRs operated using HS autograft were performed in skeletally mature patients at the author's institute between July 2007 and December 2021. Among these, 219 ACLRs were excluded, including (i) 52 patients with unknown smoking status, (ii) 141 patients who did not undergo post‐operative MRI and (iii) 26 patients who had a follow‐up of less than 2 years. Four hundred ninety‐five patients met all inclusion and exclusion criteria, including 397 males and 98 females. (Figure 1) The average age of the patients was 27.2 ± 8.2 years (range = 14–56 years). The average follow‐up was 78 ± 48 months. The longest follow‐up was 197 months. Post‐operative MRIs were performed for all included patients at an average of 20.2 ± 12.5 months after the index ACLR.

There were a total of 56 ACLR graft ruptures (11.3%) which occurred at an average of 54.9 ± 42 months after the index ACLR (range = 6–169 months). The median yearly rate of graft rupture among the whole cohort was 0.9 per 100 ACLRs per year (interquartile range [IQR] = 0.7).

There were 99 smokers, including 64 daily smokers, 19 non‐daily smokers and 16 ex‐smokers. The rate of smoking was 20%. Smokers were older than non‐smokers (*p* = 0.046). There were more men among smokers (94.9%) compared to non‐smokers (76.5%) (*p* < 0.001). The body mass index was higher in smokers (*p* = 0.028). Other than these, there were no differences between smokers and non‐smokers in terms of other demographic data, including the laterality of the injured knee, the time between the index injury and ACLR, the pre‐injury TAS, the pre‐operative IKDC subjective score, the length of follow‐up, and the time between surgery and the post‐operative MRI (Table 1).

**Table 1:** Comparison of demographic data between smokers and non‐smokers in the whole cohort.

	Smokers	Non‐smokers	*p* (Student's *t*‐test, if not specified)
Number	99	396	
Age	28.5 ± 6.9	26.9 ± 8.5	*p* = 0.046*
Sex (men vs. women)	94 vs. 5	303 vs. 93	*p* < 0.001* (chi‐square test)
Laterality (right vs. left)	48 vs. 51	213 vs. 183	*p* = 0.345 (chi‐square test)
Body mass index	24.9 ± 4.4	24.1 ± 3.4	*p* = 0.028*
Time between injury and index ACLR (days)	525 ± 839	586 ± 1178	*p* = 0.314
Pre‐injury Tegner activity scale	6.5 ± 1.1	6.4 ± 1.4	*p* = 0.281
Pre‐operation IKDC	61.5 ± 12.6	61.2 ± 14.9	*p* = 0.442
Follow‐up (months)	77 ± 52	78 ± 48	*p* = 0.472
Time between MRI and surgery (months)	20.1 ± 10.4	20.2 ± 12.9	*p* = 0.485

Abbreviations: ACLR, anterior cruciate ligament reconstruction; IKDC, International Knee Documentation Committee; MRI, magnetic resonance imaging.

* Statistical significant.

**Figure 2:**
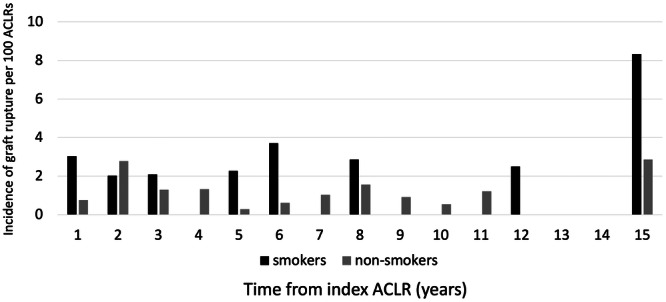
Rate of graft ruptures among smokers and non‐smokers in relation to years post‐surgery. ACLR, anterior cruciate ligament reconstruction.

A total of 16.2% of smokers, compared to 10.1% of non‐smokers, suffered from graft rupture. The yearly rate of graft rupture is presented in Figure 2. The median yearly rate of graft rupture for smokers was 2 per 100 ACLRs (IQR = 2.4), compared to 1 per 100 ACLRs for non‐smokers (IQR = 0.6) (Figure 2).

**Figure 3:**
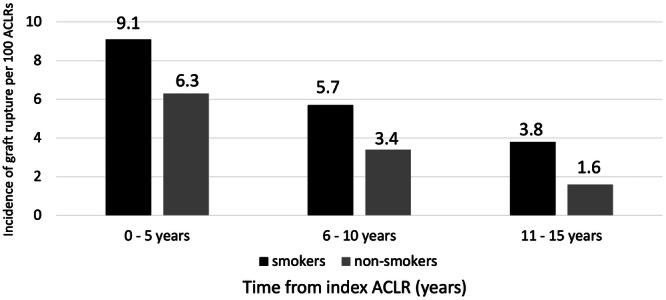
Five‐year rate of graft ruptures between smokers and non‐smokers in relation to years after surgery. ACLR, anterior cruciate ligament reconstruction.

The 5‐year rate of graft rupture was highest in the first 5 years post‐operation. There was a progressive decline in the 5‐year rate of graft rupture in both smokers and non‐smokers with increasing time from the index ACLR (Figure 3). The corresponding 5‐year rate of graft rupture in smokers was around 1.5–2.5 times that of non‐smokers, regardless of whether it was 0–5, 6–10 or 11–15 years after ACLR.

Forty‐one patients underwent revision ACLRs (8.3%). Thirteen smokers compared to 28 non‐smokers received revision surgeries.

### Subgroup analysis—Case–control matching study

One hundred seventy‐two patients were matched in a one‐to‐one ratio. All patients in the case–control matching study had a minimum follow‐up of 2 years. Apart from the smoking status, the two groups were comparable regarding the demographic data (Table 2).

**Table 2:** Comparison of demographic data between smokers and non‐smokers in the case–control study.

	Case (smokers)	Control (non‐smokers)	*p* (Student's *t*‐test, if not specified)
Number	86	86	–
Follow‐up (months)	73 ± 51	72 ± 51	*p* = 0.45
Time between MRI and surgery (months)	19.9 ± 10.4	19.7 ± 17.4	*p* = 0.47
Age	28.1 ± 7.0	27.5 ± 7.3	*p* = 0.301
Sex (men vs. women)	82 vs. 4	82 vs. 4	–
Laterality (right vs. left)	41 vs. 45	47 vs. 39	*p* = 0.36 (chi‐square test)
Body mass index	24.3 ± 3.8	24.1 ± 3.0	*p* = 0.343
Pre‐injury Tegner activity scale	6.6 ± 1.0	6.7 ± 1.1	*p* = 0.151
Time between injury and index ACLR (days)	530 ± 874	431 ± 663	*p* = 0.201
Pre‐operation IKDC	60.7 ± 12.6	59.7 ± 14.8	*p* = 0.326
KT‐1000 side‐to‐side difference at 30 lb (mm)	4.0 ± 2.6	3.7 ± 2.2	*p* = 0.236

*Note*: –: statistical comparison not performed.

Abbreviations: ACLR, anterior cruciate ligament reconstruction; IKDC, International Knee Documentation Committee; MRI, magnetic resonance imaging.

The cases and the controls were identical in the operative details, including Grade 3 pivot shift phenomenon during EUA, type of ACLR regarding single‐bundle and double‐bundle, concomitant anterolateral ligament reconstruction, and type of autograft used. The average diameter of the ACLR graft was the same in both groups (Table 3).

**Table 3:** Operative information between smokers and non‐smokers in the case–control study.

	Case (smokers)	Control (non‐smokers)	*p* (Student's *t*‐test, if not specified)
Grade 3 pivot shift test at	1%	1%	–
Single‐bundle vs. double‐bundle	58 vs. 28	58 vs. 28	–
Concomitant ALLR	17%	17%	–
Type of autograft	HS = 86	HS = 86	–
ACLR graft diameter (mm)	7.8 ± 1.1	7.8 ± 1.2	*p* = 0.473

*Note*: –: statistical comparison not performed.

Abbreviations: ACLR, anterior cruciate ligament reconstruction; ALLR, anterolateral ligament reconstruction; EUA, examination under anaesthesia; HS, hamstring.

### Graft rupture after ACLR and revision surgery in the case–control study

**Figure 4:**
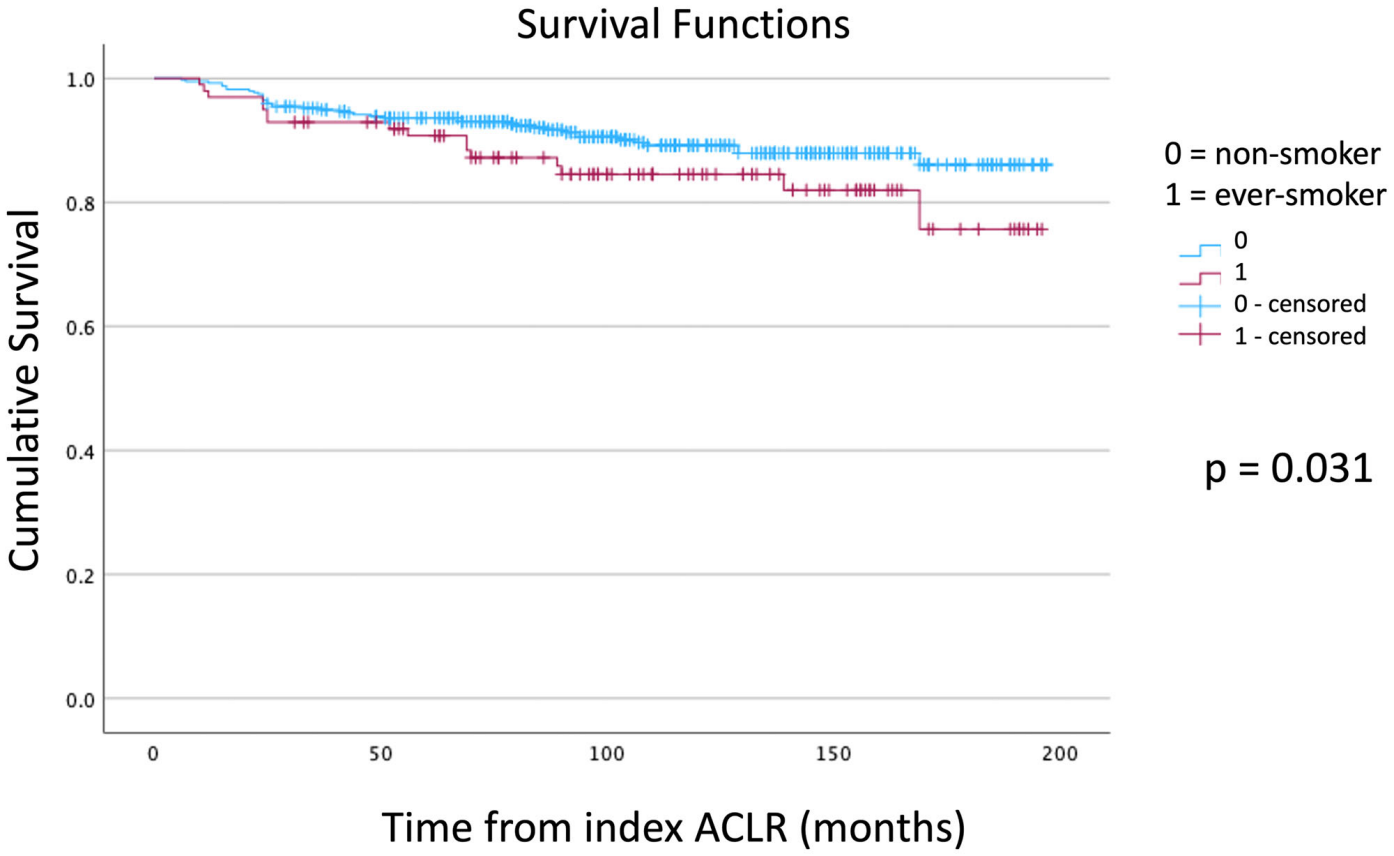
Kaplan–Meier analysis of ACLR graft rupture: Case–control matched comparison of smokers and non‐smokers. ACLR, anterior cruciate ligament reconstruction.

In the case–control matching study, smokers were found to be more prone to suffering from graft rupture after ACLR compared to non‐smokers (19% and 8%, respectively; *p* = 0.044; OR = 2.6, 95% CI = 1.01–6.63). However, there was no difference in the time of graft rupture after ACLR between the two groups (52 ± 49 and 46 ± 34 months, respectively; *p* = 0.389). The survivorship of ACLR was poorer in smokers compared to non‐smokers (*p* = 0.031, log‐rank test, Kaplan–Meier survivorship analysis) (Figure 4).

There was also no difference between the two groups in the rate of revision surgeries (*p* = 0.089).

## DISCUSSION

In a cohort of 495 primary ACLRs operated using HS autograft with a smoking rate of 20%, the rates of ACLR graft rupture and revision ACLR at a mean follow‐up of 6.5 ± 4 years were 11.3% and 8.3%, respectively. The 5‐year rate of graft rupture was highest in the first 5 years post‐operation for both smokers and non‐smokers. In a subgroup analysis adopting a one‐to‐one case–control matching methodology, smokers were found to have a higher risk of suffering from graft rupture (OR = 2.6, 95% CI = 1.01–6.63) and a poorer survivorship of ACLR (*p* = 0.031, Kaplan–Meier analysis), compared to non‐smokers.

The finding of the current study aligns with the findings of a large‐scale case–control matching study conducted by Cancienne et al., who reported that the chance of subsequent ACLR reconstruction, including both ipsilateral knee revision ACLR and subsequent contralateral knee ACLR, was 1.7 times higher in smokers than in non‐smokers [4]. Cancienne et al. defined graft failure as revision ACLR, which differs from the definition adopted in the current study. The fact that not all the patients who suffered from graft rupture underwent revision surgery might be one of the explanations for the smaller OR found by Cancienne et al. compared to the current study.

Graft rupture after ACLR is influenced by a large number of covariates [8, 12, 13, 14, 21, 25, 26, 27, 28, 30, 31, 33]. Despite being a significant risk factor [4, 20], smoking is not the most important covariate that predicts ACLR graft rupture [20]. Regarding graft rupture at short‐term follow‐up, the most important risk factors are young age [12, 14, 26], chronicity of tear [13] and pre‐operative EUA pivot shift 3 phenomenon [14]. Early return to pre‐injury contact sport [11], a diameter of an HS graft less than 8 mm [29] and female gender [31] are additional factors known to increase the chance of graft rupture. To investigate the association between smoking and ACLR graft rupture, it is crucial to control most of these covariates, either in the form of an RCT or a retrospective case–control matching study.

There were studies reporting no association between smoking and graft rupture after ACLR [1, 11, 16]. Andernord et al. [1] conducted a prospective study of 16,930 primary ACLRs using data from the Swedish National Knee Ligament Register. They concluded that there was no significant association between tobacco use and revision surgery. However, important covariates such as injury‐to‐surgery time, graft diameter, type of graft (e.g., HT vs. BPTB), and activity at the time of the injury were not controlled when comparing smokers and non‐smokers [1]. Kaeding et al. [16] conducted a study investigating the potential risk factors for ipsilateral ACLR graft retear in 2683 patients. They found that smoking status was not associated with ACLR graft rupture. A multivariable logistic regression was used to determine whether the studied variables were associated with ACLR graft rupture. However, no attempt was made to adjust for potential group differences when comparing the odds [16]. Cronstrom et al. performed a systematic review of the risk factors for graft rupture following ACLR. In terms of the association with smoking, data from five papers were pooled together. The authors reported no association between smoking and graft rupture. However, the authors also commented that the quality of the included papers was low [11]. Unlike Cancienne et al.'s publication [4] and the current research, these studies did not control the important covariates for ACLR graft rupture. This may be the reason explaining the controversy surrounding the relation of smoking with ACLR graft rupture in the literature.

It is important to discover the potential association between smoking and ACLR graft rupture. Smoking is preventable and is a potentially reversible health hazard [15]. It is different from other patient‐related risk factors for ACLR graft rupture, such as young age, female gender and familiar predisposition, which are irreversible. Additionally, the rate of smoking in patients who received ACLR is high, which is 20% in the current cohort. In an ACLR study performed by Kim et al. in South Korea, the rate of smoking even approached 40% [18]. The high prevalence of smoking in patients who received ACLR contradicts the general belief that athletes do not smoke as much as those who do not play sports because they are more health‐conscious [7]. Confirmation of a positive association of smoking with ACLR graft rupture provides additional evidence supporting the importance of anti‐smoking campaigns, especially in a subgroup of young patients who receive ACLR.

## LIMITATION

First, e‐cigarettes and heated tobacco products were not included in the definition of smoking in this study, but they have become more popular in recent years [3]. Additionally, smoking status was recorded at the time of ACLR only. There was no continuous follow‐up of the smoking habit in the post‐operative period, when there may be a change in the smoking status. Second, smokers and non‐smokers were known to have different demographic characteristics, which were also confounders affecting the ACLR graft rupture rate [9]. Although a case–control matching subgroup analysis was performed to investigate the association between smoking and ACLR graft rupture and an attempt to match most known pre‐operative and intra‐operative covariates was made, the retrospective nature of the study introduced bias into the results. Third, although all patients were scheduled to receive post‐operative MRI in the second year after ACLR, 141 out of 714 patients refused to attend the imaging session, again introducing bias to the study. Fourth, repeated post‐operative MRIs were performed after a 2‐year follow‐up for patients only when there was clinical suspicion of graft rupture or other intra‐articular injury during the subsequent follow‐up. There was a possibility that some patients developed subsequent asymptomatic graft rupture, despite the post‐operative 2‐year MRI showing an intact graft. Finally, selection bias existed in the current study. Fifty‐two patients were excluded because their smoking status was not known, introducing bias to the analysis.

## CONCLUSION

In a cohort of 495 primary ACLRs operated using HS autograft with a smoking rate of 20%, the rate of ACLR graft rupture and revision ACLR at a mean follow‐up of 6.5 ± 4 years were 11.3% and 8.3%, respectively. Smoking was associated with an increased risk of graft rupture in a case–control matching subgroup analysis.

## AUTHOR CONTRIBUTIONS

The author conceived and designed the study, prepared the materials, collected the data and analyzed the data. The author wrote the first draft of the manuscript and revised previous versions. The author read and approved the final manuscript.

## CONFLICT OF INTEREST STATEMENT

The author declares no conflicts of interest.

## ETHICS STATEMENT

The current study involves human participants. The local ethics committee (Institutional Review Board of the University of Hong Kong/Hospital Authority Hong Kong West Cluster) approved and monitored the current study (Document number: UW 24‐412).

## Data Availability

The data of this study are available from the author upon reasonable request and with permission of the local ethics committee.

## References

[1] Andernord D , Desai N , Björnsson H , Ylander M , Karlsson J , Samuelsson K . Patient predictors of early revision surgery after anterior cruciate ligament reconstruction: a cohort study of 16,930 patients with 2‐year follow‐up. Am J Sports Med. 2015;43(1):121–127. 25325560 10.1177/0363546514552788

[2] Anderson AB , Dekker TJ , Pav V , Mauntel TC , Provencher MT , Tokish JM , et al. Survival of anterior cruciate ligament reconstructions in active‐duty military populations. Knee Surg Sports Traumatol Arthrosc. 2023;31(8):3196–3203. 36809509 10.1007/s00167-023-07335-w

[3] Armstrong ML , Smith N , Tracey R , Jackman H . The orthopedic effects of electronic cigarettes: a systematic review and pediatric case series. Children (Basel). 2022;9(1):62. 35053687 10.3390/children9010062 PMC8774690

[4] Cancienne JM , Gwathmey FW , Miller MD , Werner BC . Tobacco use is associated with increased complications after anterior cruciate ligament reconstruction. Am J Sports Med. 2016;44(1):99–104. 26526974 10.1177/0363546515610505

[5] Census and Statistics Department, Hong Kong Special Administrative Region . Thematic Household Survey Report No. 36. Pattern of smoking. Available from: https://www.censtatd.gov.hk/en/data/stat_report/product/C0000047/att/B11302532013XXXXB0100.pdf. Accessed 29 Nov 2024.

[6] Census and Statistics Department, Hong Kong Special Administrative Region . Thematic Household Survey Report No. 75. Pattern of smoking. Available from: https://www.censtatd.gov.hk/en/data/stat_report/product/C0000047/att/B11302752022XXXXB0100.pdf. Accessed 11 Dec 2024.

[7] Chaabane Z , Murlasits Z , Mahfoud Z , Goebel R . Tobacco use and its health effects among professional athletes in Qatar. Can Respir J. 2016;2016:2684090. 28025593 10.1155/2016/2684090 PMC5153470

[8] Chan YC , Yau WP . Association of smoking with graft rupture after anterior cruciate ligament reconstruction. Orthop J Sports Med. 2022;10(10):23259671221127244. 36263312 10.1177/23259671221127244 PMC9575463

[9] Chung WS , Kung PT , Chang HY , Tsai WC . Demographics and medical disorders associated with smoking: a population‐based study. BMC Public Health. 2020;20(1):702. 32414354 10.1186/s12889-020-08858-4 PMC7227312

[10] Cronström A , Tengman E , Häger CK . Risk factors for contra‐lateral secondary anterior cruciate ligament injury: a systematic review with meta‐analysis. Sports Med. 2021;51(7):1419–1438. 33515391 10.1007/s40279-020-01424-3 PMC8222029

[11] Cronström A , Tengman E , Häger CK . Return to sports: a risky business? A systematic review with meta‐analysis of risk factors for graft rupture following ACL reconstruction. Sports Med. 2023;53(1):91–110. 36001289 10.1007/s40279-022-01747-3 PMC9807539

[12] Everhart JS , Yalcin S , Spindler KP . Twenty‐year outcomes after anterior cruciate ligament reconstruction: a systematic review of prospectively collected data. Am J Sports Med. 2022;50(10):2842–2852. 34591691 10.1177/03635465211027302

[13] Fältström A , Hägglund M , Magnusson H , Forssblad M , Kvist J . Predictors for additional anterior cruciate ligament reconstruction: data from the Swedish national ACL register. Knee Surg Sports Traumatol Arthrosc. 2016;24(3):885–894. 25366191 10.1007/s00167-014-3406-6

[14] Firth AD , Bryant DM , Litchfield R , McCormack RG , Heard M , MacDonald PB , et al. Predictors of graft failure in young active patients undergoing hamstring autograft anterior cruciate ligament reconstruction with or without a lateral extra‐articular tenodesis. The Stability experience. Am J Sports Med. 2022;50(2):384–395. 35050817 10.1177/03635465211061150 PMC8829733

[15] Reitsma MB , Kendrick PJ , Ababneh E , Abbafati C , Abbasi‐Kangevari M , Abdoli A , et al. Spatial, temporal, and demographic patterns in prevalence of smoking tobacco use and attributable disease burden in 204 countries and territories, 1990‐2019: a systematic analysis from the Global Burden of Disease Study 2019. Lancet. 2021;397(10292):2337–2360. 34051883 10.1016/S0140-6736(21)01169-7 PMC8223261

[16] Kaeding CC , Pedroza AD , Reinke EK , Huston LJ , Spindler KP , Amendola A , et al. Risk factors and predictors of subsequent ACL injury in either knee after ACL reconstruction: prospective analysis of 2488 primary ACL reconstructions from the MOON cohort. Am J Sports Med. 2015;43(7):1583–1590. 25899429 10.1177/0363546515578836 PMC4601557

[17] Karim A , Pandit H , Murray J , Wandless F , Thomas NP . Smoking and reconstruction of the anterior cruciate ligament. J Bone Joint Surg Br. 2006;88(8):1027–1031. 16877601 10.1302/0301-620X.88B8.17189

[18] Kim SJ , Lee SK , Kim SH , Kim SH , Ryu SW , Jung M . Effect of cigarette smoking on the clinical outcomes of ACL reconstruction. J Bone Jt Surg. 2014;96(12):1007–1013. 10.2106/JBJS.M.00598 24951736

[19] Lindqvist Bueneman S , Sernert N , Kvist J , Kartus JT . Analysis of the Swedish Knee Ligament Register: concomitant injuries, revision surgery and smoking render worse results. Knee Surg Sports Traumatol Arthrosc. 2024;32(11):2895–2908. 38869078 10.1002/ksa.12307

[20] Lu Y , Till SE , Labott JR , Reinholz AK , Hevesi M , Krych AJ , et al. Graft failure and contralateral ACL injuries after primary ACL reconstruction: an analysis of risk factors using interpretable machine learning. Orthop J Sports Med. 2024;12(10):23259671241282316. 39464204 10.1177/23259671241282316 PMC11504090

[21] Magnussen RA , Meschbach NT , Kaeding CC , Wright RW , Spindler KP . ACL graft and contralateral ACL tear risk within ten years following reconstruction: a systematic review. JBJS Rev. 2015;3(1):e3. 10.2106/JBJS.RVW.N.00052 27501023

[22] Nicholls M , Ingvarsson T , Filbay S , Lohmander S , Briem K . Smoking and secondary ACL rupture are detrimental to knee health post ACL injury—a Bayesian analysis. J Exp Orthop. 2023;10(1):79. 37556084 10.1186/s40634-023-00638-4 PMC10412518

[23] Novikov DA , Swensen SJ , Buza JA , Gidumal RH , Strauss EJ . The effect of smoking on ACL reconstruction: a systematic review. Phys Sportsmed. 2016;44(4):335–341. 27456300 10.1080/00913847.2016.1216239

[24] Roecker Z , Kamalapathy P , Werner BC . Male sex, cartilage surgery, tobacco use, and opioid disorders are associated with an increased risk of infection after anterior cruciate ligament reconstruction. Arthroscopy. 2022;38(3):948–952.e1. 34332054 10.1016/j.arthro.2021.07.025

[25] Rojas G , Perelli S , Ibanez M , Formagnana M , Ormazabal I , Monllau JC . Effect of modified Lemaire anterolateral extra‐articular tenodesis on the magnetic resonance imaging maturity signal of anterior cruciate ligament hamstring graft. Am J Sports Med. 2021;49(9):2379–2386. 34133234 10.1177/03635465211018858

[26] Runer A , Csapo R , Hepperger C , Herbort M , Hoser C , Fink C . Anterior cruciate ligament reconstructions with quadriceps tendon autograft result in lower graft rupture rates but similar patient‐reported outcomes as compared with hamstring tendon autograft: a comparison of 875 patients. Am J Sports Med. 2020;48(9):2195–2204. 32667271 10.1177/0363546520931829

[27] Salmon LJ , Heath E , Akrawi H , Roe JP , Linklater J , Pinczewski LA . 20‐year outcomes of anterior cruciate ligament reconstruction with hamstring tendon autograft: the catastrophic effect of age and posterior tibial slope. Am J Sports Med. 2018;46(3):531–543. 29244525 10.1177/0363546517741497

[28] Samuelsen BT , Webster KE , Johnson NR , Hewett TE , Krych AJ . Hamstring autograft versus patellar tendon autograft for ACL reconstruction: is there a difference in graft failure rate? A meta‐analysis of 47,613 patients. Clin Orthop Relat Res. 2017;475:2459–2468. 28205075 10.1007/s11999-017-5278-9 PMC5599382

[29] Snaebjörnsson T , Hamrin‐Senorski E , Svantesson E , Karlsson L , Engebretsen L , Karlsson J , et al. Graft diameter and graft type as predictors of anterior cruciate ligament revision. a cohort study including 18,425 patients from the Swedish and Norwegian national knee ligament registries. J Bone Jt Surg. 2019;101(20):1812–1820. 10.2106/JBJS.18.01467 31626005

[30] Svantesson E , Sundemo D , Hamrin Senorski E , Alentorn‐Geli E , Musahl V , Fu FH , et al. Double‐bundle anterior cruciate ligament reconstruction is superior to single‐bundle reconstruction in terms of revision frequency: a study of 22,460 patients from the Swedish National Knee Ligament Register. Knee Surg Sports Traumatol Arthrosc. 2017;25(12):3884–3891. 27882413 10.1007/s00167-016-4387-4 PMC5698375

[31] Tan SHS , Lau BPH , Khin LW , Lingaraj K . The importance of patient sex in the outcomes of anterior cruciate ligament reconstructions. A systematic review and meta‐analysis. Am J Sports Med. 2016;44(1):242–254. 25802119 10.1177/0363546515573008

[32] Vaudreuil NJ , Rothrauff BB , de Sa D , Musahl V . The pivot shift: current experimental methodology and clinical utility for anterior cruciate ligament rupture and associated injury. Curr Rev Musculoskelet Med. 2019;12(1):41–49. 30706283 10.1007/s12178-019-09529-7 PMC6388573

[33] Webster KE , Feller JA , Leigh WB , Richmond AK . Younger patients are at increased risk for graft rupture and contralateral injury after anterior cruciate ligament reconstruction. Am J Sports Med. 2014;42(3):641–647. 24451111 10.1177/0363546513517540

[34] Yau WP . Smokers achieved minimal clinically important difference for visual analog scale and american shoulder and elbow surgeons scores at a lower rate than nonsmokers even when repaired supraspinatus tendons were intact on postoperative magnetic resonance imaging. Arthrosc Sports Med Rehabil. 2024;6(2):100877. 38379600 10.1016/j.asmr.2023.100877 PMC10877171

